# On a Venous Pulse in the Veins on the Back of the Hand

**Published:** 1845-12

**Authors:** 


*On a Venous Pulse in the Veins on the back of the Hand.* By M. Martin-Solon.—M. Martin-Solon's paper on the venous pulse, had not reference to the pulsation seen in the jugular veins, depending on a reflux of blood from the right auricle of the heart, but to a pulsation in the dorsal veins of the hand, synchronous with that of the radial and ulnar arteries. When the veins were pressed at their origins at the fingers, so as to empty them, no pulsations were detected; but if rendered full and distended, by being compressed as they passed over the wrist, the pulsation was evident. Pressure on the radial or brachial arteries stopped the pulsation. M. Martin-Solon endeavored to show that this pulsation could not depend on some of the veins passing over minute branches of arteries, nor on any slight motion of the subjacent tendons. He found it most constantly present in persons who had been laboring under acute diseases for which they were copiously bled. He therefore attributed this venous pulse to the excessive fluidity of the blood, allowing the impulse from the arteries to be communicated to the veins through the capillaries.—*Ed. Med. Jour., from Bull. de l'Acad. Roy. de Med.*

